# Profiling, monitoring and conserving caterpillar fungus in the Himalayan region using anchored hybrid enrichment markers

**DOI:** 10.1098/rspb.2021.2650

**Published:** 2022-04-27

**Authors:** Zhengyang Wang, Wa Da, Chandra Singh Negi, Puspa Lal Ghimire, Karma Wangdi, Pramod K. Yadav, Zhuoma Pubu, Laiku Lama, Kuenga Yarpel, Sarah C. Maunsell, Yong Liu, Krushnamegh Kunte, Kamaljit S. Bawa, Darong Yang, Naomi E. Pierce

**Affiliations:** ^1^ Department of Organismic and Evolutionary Biology and Museum of Comparative Zoology, Harvard University, 26 Oxford Street, Cambridge, MA 02138, USA; ^2^ Tibetan Plateau Institute of Biology, Tibet Autonomous Region, Lhasa 850001, People's Republic of China; ^3^ Department of Zoology, M B Government Postgraduate College, Haldwani (Nainital) 263139, Uttarakhand, India; ^4^ Asia Network for Sustainable Agriculture and Bioresources (ANSAB), Baneshwor, Kathmandu, Nepal; ^5^ Ugyen Wangchuck Institute for Conservation and Environmental Research, Lamai Goempa, Bumthang, Jakar 32001, Bhutan; ^6^ Department of Parks, Recreation, and Tourism Management, Clemson University, Clemson, SC 29634-0735, USA; ^7^ Himalayan Herbs Traders, Baluwatar-4 Bagta Marga 161, Kathmandu, Nepal; ^8^ Changzeeri, Thimphu 11001, Bhutan; ^9^ Institute of Plant Protection, Sichuan Academy of Agricultural Sciences, Chengdu 610066, People's Republic of China; ^10^ National Centre for Biological Sciences, Tata Institute of Fundamental Research, Bengaluru 560065, India; ^11^ University of Massachusetts, Boston, MA 02125, USA; ^12^ Ashoka Trust for Research in Ecology and the Environment, Bangalore 560024, India; ^13^ Xishuangbanna Tropical Botanical Garden, Chinese Academy of Sciences, Mengla, Yunnan 666303, People's Republic of China

**Keywords:** caterpillar fungus trade, *Ophiocordyceps sinensis*, *Thitarodes*, phylogeny, molecular reference library, trans-boundary conservation

## Abstract

The collection of caterpillar fungus accounts for 50–70% of the household income of thousands of Himalayan communities and has an estimated market value of $5–11 billion across Asia. However, Himalayan collectors are at multiple economic disadvantages compared with collectors on the Tibetan Plateau because their product is not legally recognized. Using a customized hybrid-enrichment probe set and market-grade caterpillar fungus (with samples up to 30 years old) from 94 production zones across Asia, we uncovered clear geography-based signatures of historical dispersal and significant isolation-by-distance among caterpillar fungus hosts. This high-throughput approach can readily distinguish samples from major production zones with definitive geographical resolution, especially for samples from the Himalayan region that form monophyletic clades in our analysis. Based on these results, we propose a two-step procedure to help local communities authenticate their produce and improve this multi-national trade-route without creating opportunities for illegal exports and other forms of economic exploitation. We argue that policymakers and conservation practitioners must encourage the fair trade of caterpillar fungus in addition to sustainable harvesting to support a trans-boundary conservation effort that is much needed for this natural commodity in the Himalayan region.

## 1. Introduction

The entomopathogenic fungus *Ophiocordyceps sinensis* (Berk.) Sung, 2007 (Hypocreales: Ophiocordycipitaceae) parasitizes the larvae of moths in the genus *Thitarodes* Viette, 1968 (Lepidoptera: Hepialidae). *Ophiocordyceps sinensis* parasitizes soil-boring *Thitarodes* larvae: the fungal mycelium proliferates throughout the larval tissues and extrudes a stroma through the head capsule of its host, out of the soil surface to release ascospores. The whole complex hardens into a mummified, caterpillar-shaped bundle of fungal mycelium and stroma commonly referred to as ‘caterpillar fungus’.

This moth–fungus symbiont was first described in the 15th century by Tibetan scholars and has since been avidly collected in its endemic range by Chinese and Tibetans as an ethnomedicine [1]. Accounts from the eighteenth century suggest already well-established trade routes from historical Tibet to coastal China [2,3]. Present-day demand for caterpillar fungus from mainland China has been known to drive its price up to more than three times that of gold [4]. The collection of caterpillar fungus from the wild generates the primary source of income for hundreds of thousands of collectors [5]. Conservative estimates place annual production of dried caterpillar fungus at 100 tons [6], amounting to 300 million individual caterpillar fungi collected per year at a market value of $5–11 billion [7]. Intense territorial conflicts over land ownership and collection rights have arisen across the Himalaya and the Tibetan Plateau, the range of caterpillar fungus [8]. Such conflicts will persist as suitable habitats continue to shift and decrease due to climate change [9–11].

Although caterpillar fungus was traditionally collected only within the Chinese border of the Tibet Autonomous Region (TAR) and the provinces of Qinghai, Gansu, Sichuan and Yunnan, the past decade has seen nations on the southern slope of the Himalaya (India, Bhutan, Nepal) lured into this lucrative supply chain [12–17]. For these Himalayan communities, 50–70% of the local seasonal household income is derived from these collections, transforming local economies and reducing poverty for tens of thousands of people [18–20]. However, Himalayan collectors are at multiple economic disadvantages compared with collectors on the Tibetan Plateau that have long-established trade relations with mainland Chinese consumers. For example, strict state regulations for caterpillar fungus trade in India resulted in the annual transportation of $5–7.5 million worth of local products across the Nepalese border to be ‘legalized’ for export [13,21,22]. Moreover, a $10 million per annum export–import difference exists between the Chinese and Nepalese custom borders, suggesting the majority of ‘Nepalese’ caterpillar fungus does not clear local customs [23].

The current pattern of Himalayan caterpillar fungus trade operates in a ‘licit but illegal’ grey zone and can be summarized as (1) a cross-border legalization process from India to Nepal, followed by (2) a post-export de-origination process upon entering the Chinese border from Nepal. This provides ample opportunity for both state-actor corruption and non-state-actor exploitation that ultimately is detrimental to the economies of local communities and thwarts conservation action for the long-term persistence of caterpillar fungus populations.

Top-down policies continue to adapt and find the best practice of sustainable caterpillar fungus harvest in each region [21,22,24–27]. However, local stakeholder and governing regimes would benefit from asserting their ownership of locally available natural resources, often found on community lands [28]. Such a process could be institutionalized by recognizing rights to own, manage and use wild resources by individuals as well as local governing bodies such as tribal societies and panchayats (e.g. through the Indian Forest Rights Act, https://www.fra.org.in).

Through these institutions, communities could then prepare biodiversity registers that would make a catalogue of natural resources such as the caterpillar fungus found on community lands. This framework for local product origin authentication, as has been applied in authenticating agricultural food products (reviewed in [29]), might resolve the Himalayan caterpillar fungus trade grid-lock. From the bottom up, origin authentication puts a direct link between communities and the market, and thus begets more economic incentives to local communities to provide high-quality products [30–32]. From the top down, the ability to determine the origin of a product allows state regulators to resolve trade conflict more effectively and detect product adulteration [33–36].

DNA-based origin authentication relies on molecular techniques to obtain genetic material from samples so that they can be assigned to geographical genetic clusters with which they are most similar. In the context of the wildlife trade, these techniques have been successfully applied to trace geographical origins of ivory [37], shark fins [38], pet birds [39] and primates [40]. Since the host moths of caterpillar fungi occupy a large range of topologically complex terrain [41,42], considerable isolation-by-distance has arisen both intraspecifically [43] and interspecifically [44]. This makes an authentication system based on molecular markers to reveal the identity and origin of host moths feasible, while the *O. sinensis* fungal strains that parasitize them show comparatively less genetic differentiation (only 1% average genetic differences in fungal sequences versus 5% mean genetic difference in host moth samples, see [44]). Nevertheless, attempts to recover genetic fragments for host moths (*Thitarodes*) from caterpillar fungus using traditional Sanger sequencing has succeeded in obtaining a maximum of three genetic loci per sample [44–46], most likely due to genetic fragmentation and degradation of the host sample during the parasitization process. Sanger sequencing also requires freshly collected, well-preserved samples.

Here, we customized a commercially available hybrid-enrichment probe set originally designed for butterfly phylogenomics [47] to build a 14-gene phylogeny of caterpillar fungus hosts. Our method belongs to a class of high-throughput sequencing techniques for isolating multiple loci (referred to as sequence capture, targeted enrichment or anchored hybrid enrichment) from traditionally low DNA-yielding samples of Lepidoptera [48–50]. Our genetic placement simulations and biogeographic analyses provide strong evidence of identifiable geographical signals from individual host samples (but not their co-evolved fungal parasites). We suggest that since *Thitarodes* hosts are highly distinctive across regions, a robust host phylogeny can serve, and be iteratively improved, as a shared molecular reference ‘library’ for caterpillar fungus host origin authentication, especially for samples collected in the Himalayan regions. Based on these results, we propose a two-step procedure to help local communities authenticate their product using this hybrid enrichment probe kit, and provide suggestions for policymakers and conservation practitioners for improving this multi-nation trade route without creating opportunities for illegal export and other forms of economic exploitation. We suggest that maintaining sustainable harvest as well as the fair trade of caterpillar fungus will support the trans-boundary conservation efforts needed in the Himalayan region [10,11,51,52].

## 2. Methods

See electronic supplementary material, Methods, for detailed descriptions.

### (a) Hybrid enrichment

**Figure 1.**
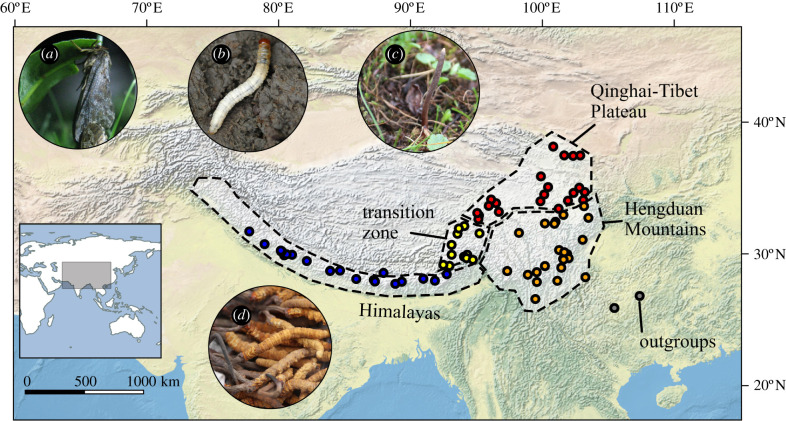
Location of caterpillar fungi used for anchored hybrid enrichment in this study. Samples are designated as being from four geographical regions across the distributional range of caterpillar fungi (dotted lines, different coloured dots are where samples were collected from different regions). Circled inserts show: (*a*) adult caterpillar fungus host (here showing *Thitarodes pui*); (*b*) larva of caterpillar fungus host (here showing *Thitarodes baimaensis*); (*c*) Fungal stroma of *O. sinensis* in the wild; (*d*) dried caterpillar fungus as sold in the market. Photo credit: Zhiwen Zou (*a*) and Darong Yang (*b*–*d*). (Online version in colour.)

We used a 13-locus target capture probe set [47] that included gene regions most commonly used for butterfly phylogenetics (see electronic supplementary material, table S1 for probe regions). We also designed a Cytb target capture probe from *Thitarodes* mitogenomes deposited in GenBank to maximize our loci overlap with existing phylogenies. We collected 94 caterpillar fungus samples from across its recorded distribution range, with 34 of these samples originating from the Himalayan regions of TAR, Nepal, Bhutan and India (figure 1 dots in blue, electronic supplementary material, table S1). All Himalayan samples were purchased from Nepalese and Bhutanese vendors with permit to export to China (samples from India were sold by Nepalese vendors and identified post-export). Samples within China are from the authors' private collection. The oldest sample is a dried caterpillar fungus that was collected in 1993. Sample DNA (a mix of *O. sinensis* and host DNA) was extracted using Qiagen DNeasy Blood and Tissue Kits. To sequence hosts, quantified DNA extracts were submitted to RAPiD Genomics (Gainesville, FL) for hybrid enrichment and sequencing following the procedure described in Espeland *et al*. [49]. The same DNA extracts were used to sequence the parasitic fungus (*O. sinensis*) using the nrDNA internal transcribed spacer (ITS) region and the Sanger sequencing protocols of Zhang *et al*. [44].

### (b) Phylogenetic reconstruction

We used an ultra-fast all-in-one FASTQ preprocessor [53] to merge subsequent raw pair-end reads, automatically detect adapters and filter low-quality reads. We used the HybPiper script v. 1.3.1 [54] to recover our targeted loci. Only samples with complete host loci recovery (all 14 loci) were used in phylogenetic reconstruction. Loci recovered from HybPiper were aligned and concatenated with MAFFT v. 7.0.1 [55]. Best model and partition schemes were estimated using ModelFinder [56]. We searched for the most likely tree topology in IQ-TREE 2.0 [57], with 1000 iterations for ultrafast bootstraps [58]. We repeated this 500 times and calculated the Robinson–Foulds distance among the most likely trees from each run to check whether the tree topology had reached a global optimum on the likelihood surface. We also inferred a species tree using a multispecies coalescent model in ASTRAL-III v. 5.7.7 [59] to account for possible incomplete lineage sorting. Each gene tree used as input for the species tree was inferred in IQ-TREE 2.0 [57] as described above, with quartet support [60] as branch support. Since it is likely that many samples represent the same host species, we conducted species delimitation analysis on our phylogeny using both a Poisson tree processes (PTP) model [61] and a general mixed Yule coalescent (GMYC) model [62].

### (c) Sensitivity tests

We visualized the increase in phylogenetic resolution using markers from additional loci by bootstrapping possible sequence alignments generated using less than 14 loci. We then assessed the likelihood of correctly identifying a caterpillar fungus from the Himalayan region using both a phylogenetic placement approach and a maximum-likelihood approach: first, we applied a parallel evolutionary placement algorithm (EPA-ng, [63]) to query sequences of Himalayan samples from this study (using from 1 to 14 loci), and calculated the confidence level of the correct placement. This simulates the process of authenticating a product from a local community when samples from that region have been incorporated into a molecular phylogeny (see first step proposed Discussion 4.1). Second, we compiled single locus host sequences deposited on GenBank by previous researchers (‘unidentified’ samples, electronic supplementary material, table S4), and incorporated them in the maximum-likelihood phylogeny while using the most likely tree obtained in 2.2 as a topological constraint. An ‘unidentified’ sample was considered to be of Himalayan origin if it was nested within or was sister to a known ‘Himalayan clade’ on the phylogeny. Back-referencing the geographical origin of these samples (as labelled in GenBank) allowed us to estimate the accuracy of using Himalayan-based monophyly to assign an unidentified sample to a region. This approach simulates the process of identifying Himalayan caterpillar fungi that have not already been catalogued in a molecular phylogeny, similar to a market survey where regulators need to ascertain the origin of unknown samples (see second step proposed in Discussion 4.2).

### (d) Biogeographic and cophylogenetic signals

We used BioGeoBEARS [64] to infer dispersal history and ancestral range of caterpillar fungus hosts from our phylogeny. We designated samples to four geographical regions: (1) Qinghai–Tibet Plateau, (2) Hengduan Mountains, (3) the Himalaya and (4) a transition zone between the western Hengduan Mountains and the eastern Himalaya (figure 1, see electronic supplementary material, Methods, for rationale of region designation). We compared the likelihood of models of species dispersal with different emphasis on anagenetic and cladogenetic events [65]. We then performed biogeographical stochastic mapping [66] as implemented in BioGeoBEARS [64] and phytools [67] to study the historical transitions between geographical regions.

We used the ParaFit test [68] and the Procrustean Approach to Cophylogeny test (PACo; [69]) to detect signatures of co-cladogenesis between *Thitarodes* hosts and their *Ophiocordyceps* parasites. A fungal phylogeny was constructed in IQ-TREE using ITS sequences from each sample, with the multi-locus fungal phylogeny of [44] as a topological constraint. We tested the signature of co-cladogenesis among hosts and fungi at both the species-level (as delimitated in 2.2) and the individual sample level. To avoid uncertainty in phylogenetic reconstruction (especially from the fungal phylogeny), we also directly compared the matrices of sequence distances between hosts and their parasites using Mantel tests [70]. Similarly, to understand the effects of geographical isolation on the genetic differences of hosts (isolation-by-distance, IBD, [71]), we constructed matrices comparing sample locations based on (1) Euclidean geographical distances, (2) climatic differences (from WORLDCLIM 2.0, mean temperature of the coldest quarter, [72]) and (3) landscape resistance distances [73] and computed their correlation with sample genetic distances.

We further investigated the geographical ‘width’ and phylogenetic ‘depth’ of any detected IBD and cophylogenetic signals to gauge whether relationships are stronger at regional or global levels, and at historical or contemporary timescales. To do this, we first conducted a hierarchical clustering [74] of our samples based on their geographical distances (from 100 to 2000 km, at 100 km intervals), and calculated both the cophylogenetic and IBD signal of each cluster using the Mantel test. Secondly, we took synchronic ‘time slices’ of the host phylogeny and calculated both the cophylogenetic and IBD signal of each time-sliced phylogeny.

## 3. Results

Our 94 samples obtained on average 496 k reads per sample (s.d. = 451 k), 29.9% of which hit the targeted regions (s.d. = 0.16). An average sample yielded 13.2 out of the 14 genes (94% success rate), with dried samples collected as long ago as 1993 successfully yielding all 14 genes (electronic supplementary material, table S1). The only significant predictor of enrichment success rate was the year in which a sample was collected (electronic supplementary material, table S2). However, PCR and Sanger sequencing of the fungal ITS region only recovered 58 fragments out of 94 samples due to DNA degradation.

**Figure 2.**
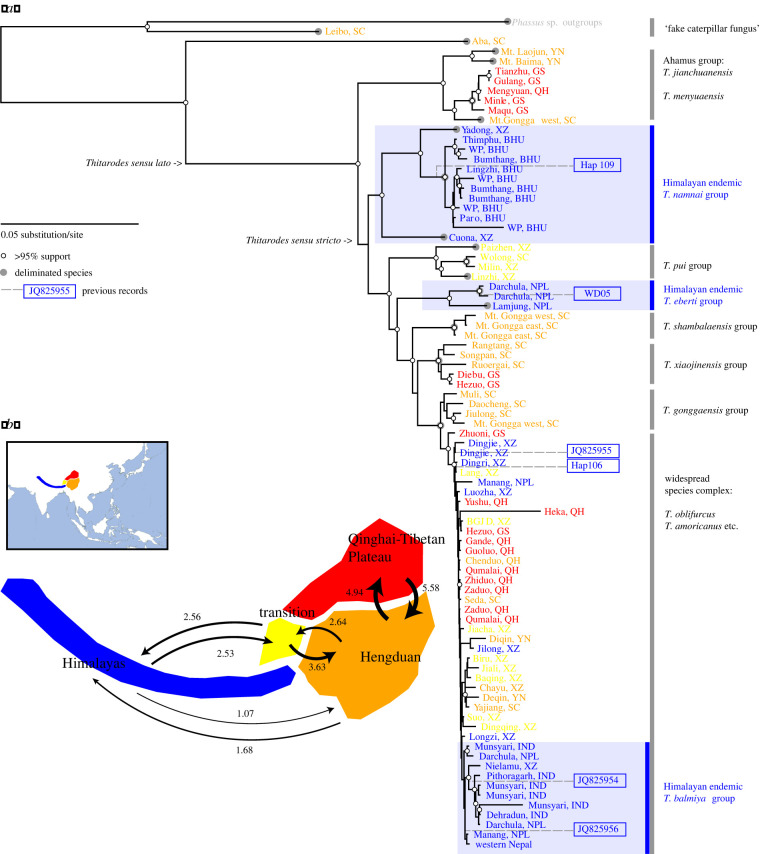
Phylogenetic and biogeographic patterns of caterpillar fungus hosts. (*a*) A 14-locus maximum-likelihood tree for all caterpillar fungi hosts in this study. Each sample is colour-coded by its sample region corresponding to (*b*). Three monophyletic groups of Himalayan samples are shaded in light blue. Blue boxes to the right of the phylogeny indicate the placement of known Himalayan samples from previous studies (identified using COI ‘barcode’ reference number in GenBank). Annotated species/clade names correspond to known taxonomic studies and are discussed in the electronic supplementary material, Discussion. (*b*) Mean number of changes in distribution ranges across 3000 stochastic character maps based on the ML phylogeny. Arrows indicate the direction of the change. Only mean changes larger than 1 are shown. (Online version in colour.)

A total of 90 out of the 94 samples achieved complete 14-loci target recovery and were used in ML tree reconstruction. IQ-TREE yielded a well-supported tree that is consistent with the coalescent-based species tree obtained using ASTRAL (electronic supplementary material, figures S1 and S2). Results from the PTP and GMYC species delimitation models were consistent (electronic supplementary material, table S3) and recovered a wide-ranging species complex from the Himalaya to the Qinghai–Tibet Plateau (figure 2*a*). The delimitations reveal at least 20 valid species of *Thitarodes* as caterpillar fungus hosts, some of which are still being taxonomically identified and described (e.g. [75]). Himalayan samples form three monophyletic clusters. Even within the unresolved single species group (figure 2*a* ‘widespread species complex’), most endemic monophyletic clusters are still highly supported.

Our simulated alignment datasets show that increasing the number of loci used for phylogenetic reconstruction significantly increased the resolution of the molecular phylogeny (electronic supplementary material, figures S3 and S4). The phylogenetic placement test that simulates the origin-authentication of already-catalogued caterpillar fungus samples indicated that they can be identified with close to 100% certainty when the reference molecular phylogeny is constructed from more than seven loci (electronic supplementary material, figure S5). With 14 loci per sample in the phylogeny, once a region's sample has been genetically catalogued, we can correctly place samples with those from the same region.

In simulating the identification of Himalayan caterpillar fungi that have not already been catalogued in a molecular phylogeny, all six cytochrome oxidase I (COI) haplotypes from GenBank were correctly placed within the Himalayan monophylies (figure 2*a*). However, one out of 10 known non-Himalayan samples was misplaced within Himalayan clusters (electronic supplementary material, figure S3, false positive).

Our best model identified the ancestral caterpillar fungus host in the Hengduan Mountains (electronic supplementary material, figures S7 and S8); all models incorporating parameters of founder event speciation (+J parameters) obtained higher likelihood compared with their counterparts without this parameter (electronic supplementary material, table S5). Species-level biogeographical stochastic mapping (BioGeoBears) shows high levels of dispersal between adjacent geographical regions (electronic supplementary material, table S6). The same pattern was recapitulated in sample-level stochastic mapping (phytools, figure 2*b* and electronic supplementary material, figure S7).

**Figure 3.**
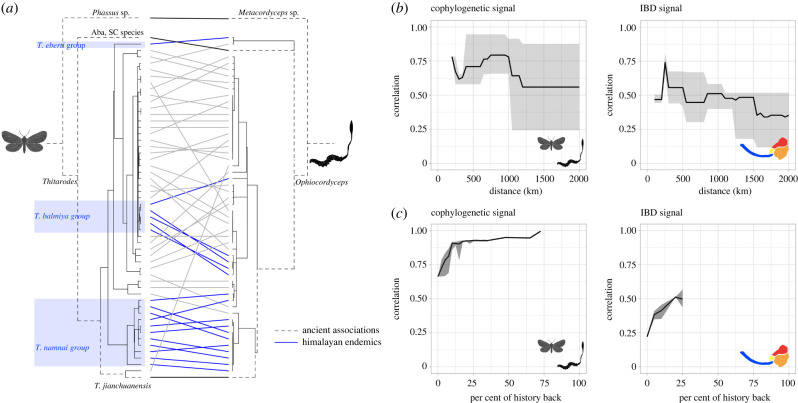
Cophylogenetic and geographical signals of caterpillar fungus hosts. (*a*) Signals of cophylogeny between caterpillar fungus hosts (left) and parasites (right) persist on more ancient lineages (dotted lines on phylogeny, connected with black lines), while groups of monophyletic Himalayan endemic hosts (blue shade) do not have co-evolved parasites. (*b*) Signals of cophylogeny and isolation by distance (IBD), as measured by significant Mantel correlations, across geographical ranges. Dark lines show the mean, and the shadows show the maximum and minimum value from all sample geographical clustering. (*c*) Signals of cophylogeny and IBD across phylogenetic depths. (Online version in colour.)

We detected significant signals of co-cladogenesis between hosts and fungal parasites (electronic supplementary material, table S8), although the strongest signals come from Hengduan Mountain samples that occupy less derived branches (figure 3*a* dotted lines, electronic supplementary material, table S8B). Monophyletic Himalayan hosts do not correspond to monophyletic groups of fungal parasites (figure 3*a*, blue lines) but DNA distances among hosts are highly correlated with those among parasites (Mantel *R* = 0.67, *p* = 0.001). Among hosts, IBD was best explained by landscape resistance distance (Mantel *R* = 0.10, *p* = 0.24) rather than climatic differences or Euclidean geographical distances among hosts (electronic supplementary material, table S9). Across hierarchical distance clusters, the greatest signal of IBD was observed in samples within 500 km of each other (figure 3*b*, right panel), and not traceable to more ancient lineages (figure 3*c*, right panel). Signals of cophylogeny were best conserved within samples approximately 1000 km of each other (figure 3*b*, left panel) and were more prominent in ancient lineages (figure 3*c*, left panel).

## 4. Discussion

**Figure 4.**
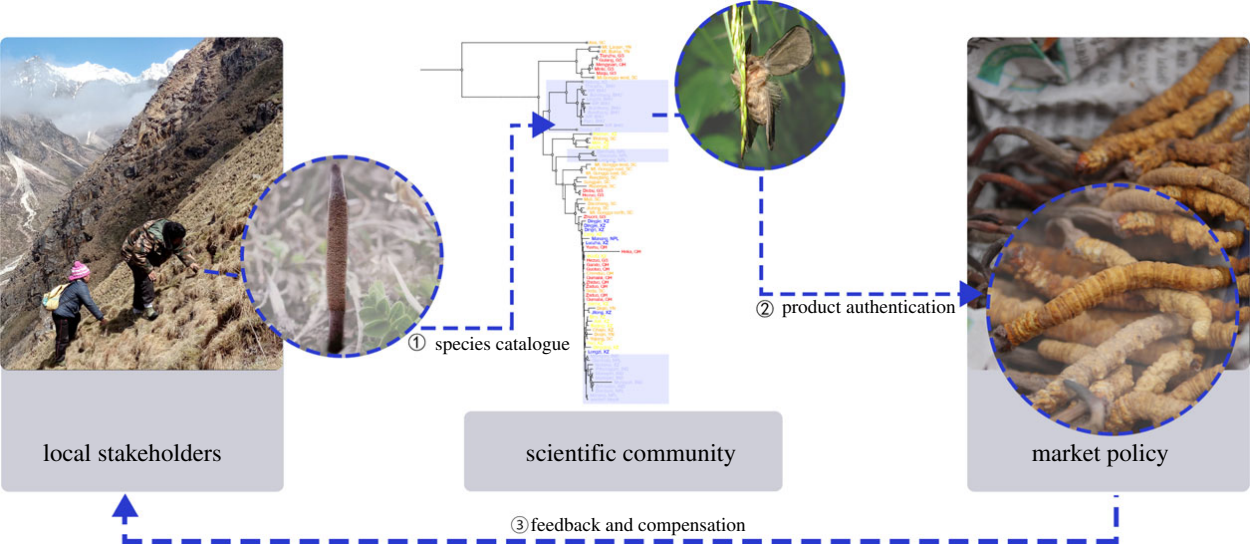
Proposed framework for using a shared molecular phylogeny to improve product origin authentication. We suggest building a molecular reference library (species catalogue) of all *Thitarodes* hosts using samples from major caterpillar fungus collection regions (step 1). We can then use this shared information to trace sample origin and authenticity in the market (step 2). This enables communities with authentic products to be recognized in the market and given proper economic compensation. Photo credit: Zhengyang Wang (left 1), Darong Yang (left 2, 4), Guren Zhang (left 3). Panel design: Yameng Huang. (Online version in colour.)

We show that an anchored hybrid enrichment probe set can recover multi-locus information for *Thitarodes* hosts, even those derived from dried market caterpillar fungi collected three decades ago. Our 14-loci phylogeny is consistent with previous three-loci phylogenies [44,45] but showed significantly better geographical resolution that was sufficient for sample origin authentication. The taxonomic and macro-evolutionary implications of our results are discussed in the electronic supplementary material, Discussion. Here we focus on the conservation of caterpillar fungus and propose a two-step procedure using a hybrid enrichment multi-locus phylogeny to empower local communities (figure 4).

### (a) Using the molecular phylogeny as a library

First, we suggest building a molecular reference library of *Thitarodes* hosts using phylogenetic data from samples representing all major caterpillar fungus collection regions. In this study, only 94 out of the 400 caterpillar fungus collection regions tallied in Hopping *et al*. [4] were sequenced. The current cost for generating multi-locus information using the hybrid enrichment protocol described here is less than $100 per sample, and a few samples per region are necessary to build a library; this cost could be integrated into a government's preparation of biodiversity registers (such as the People's Biodiversity Register in India, or the Chinese National Specimen Information Infrastructure). Clear geography-based historical dispersal (across adjacent regions, figure 2*b*), as well as significant isolation-by-distance across all landscape scales (figure 3*b*) of caterpillar fungus hosts means samples from major production zones can be readily distinguished from one another. This is especially true for samples from the Himalayan region, which have likely undergone multiple, independent dispersal events from the Hengduan Mountains (figure 2*b*; electronic supplementary material, tables S6 and S7).

At the same time, we advocate creating a molecular library of adult *Thitarodes* host species from each region by engaging with local scientists. There is a major disconnect between caterpillar fungus-derived *Thitarodes* phylogeny and descriptions of adult forms deposited in museums (reviewed in [76]). Our approach can provide a valuable link between existing museum types and caterpillar fungus forms. Within the Himalayan nations, only eight *Thitarodes* species have been recorded in Nepal [77], two in Bhutan [78] and one in India [75]. Even in the most productive collection site of caterpillar fungus in Nepal (Darchula district), the host *Thitarodes* species remains unknown. This reflects a lack of taxonomic effort (and training) rather than a lack of species diversity [79].

### (b) Origin authentication based on molecular phylogeny

Secondly, as the molecular reference library of origin-authenticated *Thitarodes* expands, conservation practitioners (or regulatory agencies) can use it to trace sample origin and authenticity in the market with as little as a single COI fragment. This ensures communities with authentic products are recognized in the market and given proper economic compensation through market consumer choice (i.e. a ‘fair trade’ model, [80]). Phylogenetic tools have already been used to detect fake ‘caterpillar fungus’ in markets, such as those using plant roots to fake fungal ascomata [81]. Now with genetic markers that facilitate finer scale identification, such detection achieves relevancy at a regional scale (see electronic supplementary material, Discussion, for market classification of regional caterpillar fungus varieties). We show that even with single COI fragments not included in our library (simulating a market survey of potentially uncatalogued samples), all six COI fragments and haploid types of known Himalayan origin could be successfully placed either within or as a sister group to known Himalayan samples (figure 2*a*, boxed labels). Once a shared library is in place (see 4.1), the cost of authenticating a sample's origin using Sanger-based genetic fragments is relatively small.

### (c) Platform for Himalayan trans-boundary conservation

The two proposed steps form a positive feedback loop between the market and communities. Origin-authenticated products allow the community to assert their fair economic contribution and enable regulators to detect smuggling. These increased economic incentives would encourage more communities to sequence their products, thereby increasing the coverage and robustness of the shared library. Cataloguing regional caterpillar fungus hosts (step 4.1) and identifying market samples (step 4.2) require operational molecular laboratories that have the capacity to conduct NGS sequencing (in the former case) or Sanger sequencing and PCR (in the latter case). Such facilities are readily available in major cities in India and China, and to a certain extent, in Nepal. Although these facilities are not located within the communities that would benefit from them, caterpillar fungi are easily transportable from the field to the lab. Multiple national agencies will need to work together to ensure caterpillar fungus authenticity for the benefit of their stakeholders, which involves sharing research facilities, standardizing sequencing pipeline and making results transparent. Our concern is that if the caterpillar fungus trade, especially in the Himalayan region, continues to operate in a legal grey zone, hundreds of local communities risk vulnerability to economic exploitation.

Caterpillar fungus trade is an economic tie that connects tens of thousands of stakeholders across international boundaries. The methods outlined here offer policymakers and conservationists a means to promote trans-boundary conservation [10,11,82]. Wang *et al*. [83] have shown that *O. sinensis* are also plant endophytic fungi that are highly reliant on alpine vegetation. Thus, sustainable harvest of caterpillar fungus also ensures preservation of alpine habitats that are extraordinarily rich in biodiversity and are situated near the source of Asia's largest rivers. Running through these habitats are international borders and sites of armed conflicts rooted in cultural misunderstanding. These habitats must be sustained for the benefit of their wild species and the many people in some of Asia's largest countries whose livelihoods depend upon them [51,52].

## Data Availability

See electronic supplementary material for additional methods and discussion, as well as sample information [84]. Raw sequences, processed sequences for phylogenetic analysis and tree files are available from the Dryad Digital Repository: https://doi.org/10.5061/dryad.msbcc2g10 [85].
